# Cardiovascular Manifestations of Multisystem Inflammatory Syndrome in Children (MIS-C) Associated With COVID-19

**DOI:** 10.7759/cureus.41950

**Published:** 2023-07-16

**Authors:** Chika C Oragui

**Affiliations:** 1 Pediatrics, Maimonides Medical Center, New York, USA

**Keywords:** multisystem inflammatory syndrome in children (mis-c), severe acute respiratory syndrome coronavirus-2 (sars-cov-2), covid-19, pediatric intensive care/intensive care, cardiovascular disease

## Abstract

Since 2019, the global pandemic caused by the SARS-CoV-2 virus, also known as COVID-19, has dramatically affected every aspect of health and society. With wide-ranging socio-economic ramifications and the morbidity/mortality associated with the disease, a lot of research has been done on this disease. With recent surges and new variants of the COVID-19 virus, we must have regularly updated information on this disease to effectively manage this disease and to maximize outcomes for patients. Worldwide data, so far, has suggested that children have milder or asymptomatic acute infectious phase, most often presenting with mild upper respiratory infection (URI) symptoms compared to the adult population. However, in the post-acute phase, it was observed that children presented with a syndrome that strongly resembled Kawasaki’s disease (KD), and like in KD, they could potentially develop severe life-threatening complications. The significant difference between KD and this syndrome is the association with COVID-19 infection. This syndrome was observed to affect almost all organ systems including cardiovascular, gastrointestinal, and integumentary and was later named multisystem inflammatory syndrome in children (MIS-C) by the Pediatric Intensive Care Society in April 2020. The cardiovascular manifestations of this clinical entity have been associated with significant morbidity and mortality. This review is an attempt to give consolidated information from the studies done so far about the cardiac changes that occur from SARS-CoV-2 infection/MIS-C.

## Introduction and background

According to the Centers for Disease Control and Prevention (CDC) [1], a multisystem inflammatory syndrome in children (MIS-C) can be defined using the following four criteria: (1) an individual aged <21 years presenting with fever, laboratory evidence of inflammation, and evidence of clinically severe illness requiring hospitalization; (2) with multisystem (≥2) organ involvement (cardiac, renal, respiratory, hematologic, gastrointestinal, dermatologic, or neurological); (3) no alternative plausible diagnoses; and (4) positive for current or recent SARS-CoV-2 (COVID-19) infection by reverse transcription-polymerase chain reaction (RT-PCR), serology, or antigen test or COVID-19 exposure within four weeks before the onset of symptoms.

The World Health Organization (WHO) case definition has six criteria: (1) children and adolescents 0-19 years of age with fever for >3 days; (2) either two of the following: (A) rash or bilateral non-purulent conjunctivitis or mucocutaneous inflammation signs (the mouth, hands, or feet); (B) hypotension or shock; (C) features of myocardial dysfunction, pericarditis, valvulitis, or coronary abnormalities (including echocardiogram {ECHO} findings or elevated troponin/N-terminal prohormone of brain natriuretic peptide {NT-proBNP}); (D) evidence of coagulopathy (by prothrombin time {PT}, partial thromboplastin time {PTT}, and elevated D-dimers); and (E) acute gastrointestinal problems (diarrhea, vomiting, or abdominal pain); (3) elevated markers of inflammation such as erythrocyte sedimentation rate (ESR), C-reactive protein, or procalcitonin; (4) no other obvious microbial cause of inflammation, including bacterial sepsis and staphylococcal or streptococcal shock syndromes; and (5) evidence of COVID-19 (RT-PCR-, antigen test-, or serology-positive) or likely contact with patients with COVID-19 [2].

## Review

Aim

The aim of the study is to summarize and categorize the changes in the cardiovascular system that occur in the pediatric population associated with SARS-CoV-2 infection/multisystem inflammatory syndrome in children (MIS-C) associated with COVID-19 and to report documented management approaches and follow-up in MIS-C with cardiovascular involvement.

Method

**Figure 1:**
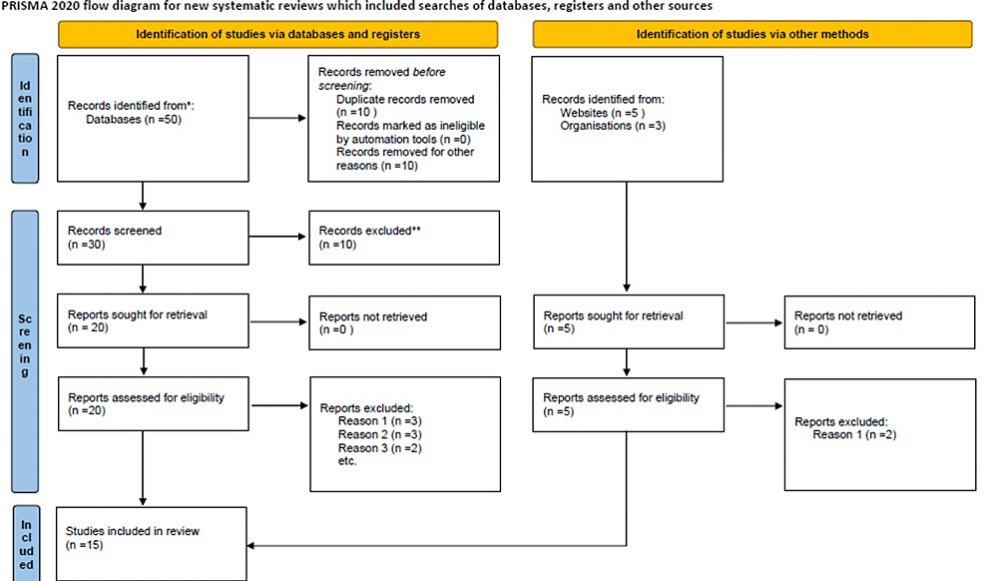
PRISMA 2020 flow diagram for systematic reviews Page MJ, McKenzie JE, Bossuyt PM, et al.: The PRISMA 2020 statement: an updated guideline for reporting systematic reviews. BMJ. 2021, 372:n71. 10.1136/bmj.n71 [3] PRISMA: Preferred Reporting Items for Systematic Reviews and Meta-Analyses

Multiple searches were made in PubMed and Google Scholar using the search terms “COVID-19,” “pediatrics,” “cardiovascular involvement,” “multisystem inflammatory syndrome,” and “MIS-C.” Articles were screened using the Preferred Reporting Items for Systematic Reviews and Meta-Analyses (PRISMA) 2020 checklist [3]. Also, data and guidelines from the websites of the Centers for Disease Control and Prevention, the World Health Organization (WHO) [1,2], and the American Heart Association (AHA) were reviewed and referenced for this literature review (Figure 1).

Pathogenesis

The pathogenetic mechanism of MIS-C has not been fully established in the literature. The most common hypothesis for this syndrome is postulated to be a postinfectious cytokine-mediated hyper-inflammatory process triggered by COVID-19 infection [4]. Some studies postulate that an antibody or T-cell recognition of self-antigens (viral mimicry of the host) results in autoantibodies formation that ultimately leads to the production of immune complexes causing damage to host cells and tissues [4]. The pathophysiologic mechanisms of cardiac insult from COVID-19 were hypothesized to be multifactorial including direct viral myocardial damage, hypoxia, hypotension, increased inflammatory status, angiotensin-converting enzyme 2 (ACE2) receptor downregulation, drug toxicity, and endogenous catecholamine adrenergic status [5]. Many of the hypotheses of the pathogenesis of cardiac complications were based on the prevailing understanding of the severe acute respiratory syndrome coronavirus (SARS-CoV), Middle East respiratory syndrome-related coronavirus (MERS-CoV), and H1N1 influenza [6].

Results

A total of 14 articles were reviewed. Some of the publications reviewed for this report used a variety of study designs including case reports, case series, observational cohort studies, and literature reviews. The common changes seen in the cardiovascular system related to MIS-C for simplification can be classified under groups including laboratory, radiographic, and clinical/symptomatic changes (Table 1).

**Table 1:** Selected articles highlighted in the table showing the cardiovascular complications associated with COVID-19 *Jiang et al. [7] included arrhythmias and LVEF dysfunction in their analysis **Choi et al. [8] and Clark et al. [10] only reported the mean levels of laboratory values rather than the population studied ProBNP, prohormone of brain natriuretic peptide; BNP, brain natriuretic peptide; LVEF, left ventricle ejection fraction

	Non-specific ECG changes	Arrhythmias	Myocardial dysfunction	Coronary artery dilatation	Pericardial effusion	ProBNP/BNP	Troponin
Rodriguez-Gonzalez et al. [4]	27%	---	52%	15%	---	73.6%	86.8%
Kochi et al. [5]	---	16.7%	---	---	---	---	16.7%
Sperotto et al. [6]	---	7%-60%	35%-100%	6%-24%	---	50%-100%	7%-60%
Jiang et al. [7]		63.3%*	63.3%*	8.9%	---	84.9%	80.9%
Choi et al. [8]	---	19%	---	---	---	**	**
Dionne et al. [9]	56%	20%	60%			44%	8%
Clark et al. [10]	19%	9%	64%	--	22%	**	**

Laboratory changes

Elevated proBNP and Brain Natriuretic Peptide (BNP)

The N-terminal prohormone of BNP (NT-proBNP) is a non-active prohormone that is released from the same molecule that produces BNP. Both molecules are typically released in response to pressure changes inside the heart, which can occur with myocardial injury. BNP elevation was more common in patients with cardiovascular involvement than cardiac troponin (cTn), occurring in 71%-100% of the cases [4]. These findings are like those reported by Sperotto et al., who reported an increase in the incidence rate of BNP and proBNP in MIS-C patients occurring anywhere from 0% to 100% in a systematic review of case reports and series from March to June of 2020 [6]. Very few studies reported no elevation in BNP. In their study, Jiang et al. reported elevated BNP rates in the patients with cardiac involvement with MIS-C of 84.9%, which is slightly higher than troponin elevation (80.9%) [7].

Elevated Troponin

All studies investigating cardiac changes associated with MIS-C reported some elevation in troponin. According to their systematic review of multiple studies in six countries published in the World Journal of Clinical Cases, Rodriguez-Gonzalez et al. [4] reported elevations in troponin ranging from 50% to 100% of the cases in some countries. A few studies showed no recorded elevation (0%) in cardiac troponin (cTn). In their study of 58 children diagnosed with MIS-C, Whittaker et al. [11] found troponin elevations in about 68% of children with MIS-C. These patients had clinical or radiological evidence of cardiac involvement associated with MIS-C [6,7].

Radiographic changes

ECG Changes

In Rodriguez-Gonzalez et al.’s [4] systematic review published in the World Journal of Clinical Cases using data from six countries, the United States, the United Kingdom, Italy, Spain, and France, they found ECG changes in MIS-C patients ranging from 3% to 97% with a mean of 22% of the patients with MIS-C cardiac involvement.

Non-specific ECG changes: Many children with a cardiac manifestation of MIS-C do have ECG changes. Like in myocarditis, these ECG changes are non-specific. In one international study of 55 patients with MIS-C, 38% had ECG abnormalities including T-wave changes, ST-segment abnormalities, and corrected QT (QTc) prolongation. Clark et al. [10] reported that 11% of their patients were reported to have an arrhythmia, including atrioventricular (AV) block, transient second-degree AV block (AVB), sinus pause, ventricular tachycardia, and idioventricular rhythm. A single-center retrospective study of 32 pediatric patients showed that 19% of these patients had first-degree AVB, occurring around eight days of initial symptoms and lasting three days with the resolution of symptoms [8].

Arrhythmias: Another complication of MIS-C/COVID-19 infection is arrhythmia. Possible pathogenetic pathways explored by some studies postulated that atrial or ventricular fibrosis secondary to myocardial injury from viral myocarditis increases the risk of arrhythmias. The incidence rate for arrhythmias varies widely with some studies reporting incidence rates from 7% to 60% [10]. Kochi et al. [5] highlighted the possibility of an increased risk of arrhythmias due to the use of hydroxychloroquine, which has been shown to induce QT prolongation.

Echocardiographic Changes

Myocardial dysfunction: Changes seen in the heart using echocardiography included myocardial dysfunction; coronary artery dilatation; and in rare cases, shock. In their multinational study, Clark et al. reported that 64% had myocardial dysfunction defined as left ventricle ejection fraction (LVEF) of <60% on echocardiography, and among these patients, 51% had mildly decreased LVEF defined as ejection fraction (EF) of 51%-60%, 32% had moderately decreased LVEF (41%-50%), and 17% had severely decreased LVEF defined as EF of <40%, with only 18% having coronary artery dilatation. Echocardiography criteria varied among the studies as some countries defined myocardial dysfunction as LVEF of <55%, whereas in the United States, and LVEF of <60% is used to define ejection fraction dysfunction [10].

Coronary artery dilatation: All the studies in this review used a similar definition for coronary artery dilatation, which is defined as coronary artery z score of >2 but <2.5 mm. All the z scores in these studies were calculated using the Boston formula. Across the board, coronary artery dilatation was classified as small for z scores of >2.3 to <5 mm, medium if the z score was >5 to <10 mm, and large or giant if the z score was >10 or >8 mm (absolute measurement in diameter). Reports on coronary artery dilatation varied across the board, with some studies showing an incidence rate of 7%; other reports were about 24% with the highest reported incidence in a UK study at 93%. Most coronary artery dilatations were mild, and giant dilatations or aneurysms were relatively rare [7-10].

Other ECHO findings include pericardial effusions, mitral regurgitation, valvular insufficiency, and regional wall abnormalities. These typically had a very low prevalence rate of about 3%.

Clinical presentation

As stated above, the patients had features that overlapped with Kawasaki’s disease (KD) with the presence of COVID-19. In cases where there was severe LVEF dysfunction, some patients presented with shock. The symptoms of MIS-C develop 2-3 months after infection, but no clear timeline has been established. Symptoms can be non-specific from fatigue, malaise, headaches, and many of the features listed in the diagnostic criteria above. Cardiac symptoms may vary as older children with more developed language may be able to explain palpitations while younger children may use words such as “butterfly” to report the subjective sensation of palpitations [11-13]. Other symptoms can be chest pain and new-onset mucosal bleeding indicating possible disseminated intravascular coagulation (DIC) [14]. Overall, mortality was exceptionally low and occurred in resource-limited countries, with very few patients presenting with severe shock with LVEF of 30% or less.

Management and follow-up

The patients presenting with shock were managed in the ICU with many requiring ionotropic support [4-11] and the use of smaller fluid boluses (10 mg/kg). The use of extracorporeal membrane oxygenation (ECMO) was rare and occurred when ionotropic support failed. The highest rates of ECMO use in MIS-C were reported by Sperotto et al. [6] to be about 28%.

Mostly, the patients presenting with MIS-C were managed in a similar modality to KD. Treatment protocols were typically institutionally guided. Intravenous immunoglobulin (IVIG) was typically used in resource-rich environments compared to resource-limited environments. The inverse was seen in steroid use with the areas of restricted resources favoring steroid use compared to the former. There was no data found comparing the outcomes of the patients who got either therapy. A combination of both IVIG and steroids was done with reported rates of 8%-64%.

Anakinra was recommended for the patients unresponsive to IVIG and steroids [15]. It is safe for children.

Given the increased risk of thrombotic complications [14], platelet suppression was typically achieved using aspirin. In many institutions including ours, aspirin was started at higher doses in the acute phases and reduced to a low dose for another six weeks with pediatric cardiology follow-up.

Sperotto et al. [6] suggested a follow-up of the patients with MIS-C and cardiac complications in 7-10 days, 4-6 weeks, 4-6 months, and 9-12 months. With repeat inflammatory markers in the immediate post-acute phases and serial ECHOs on subsequent cardiology visits, they also recommended exercise restrictions, two weeks if there is no evidence of cardiac involvement and six months if there is evidence of involvement.

While no AHA guidelines have been made specific for cardiac follow-up, the American College of Rheumatology recommends that all children with MIS-C undergo repeat echocardiograms at a minimum of 7-14 days and then 4-6 weeks after the initial presentation. For those patients with cardiac involvement noted during the acute phase of illness, another echocardiogram one year after MIS-C diagnosis could be considered [15].

## Conclusions

Cardiovascular changes seen in COVID-19 infection and MIS-C are varied and can have different ramifications. These changes are evidenced by the elevation of cardiac biomarkers, ECG, ECHO, and clinical manifestations. Despite multiple reports suggesting a complete or near-complete recovery in the pediatric population from cardiovascular injury associated with COVID-19/MIS-C, there is still an intrinsic risk of myocardial scarring in COVID-19 infection. Arrhythmias have been shown to develop because of myocardial scarring, so there is a need for studies to be done on the prognosis of the patients with cardiac complications from COVID-19/MIS-C and the possibility of these patients developing future cardiac complications.
